# Arsenic Metabolism by Human Gut Microbiota upon *in Vitro* Digestion of Contaminated Soils

**DOI:** 10.1289/ehp.0901794

**Published:** 2010-03-26

**Authors:** Tom Van de Wiele, Christina M. Gallawa, Kevin M. Kubachk, John T. Creed, Nicholas Basta, Elizabeth A. Dayton, Shane Whitacre, Gijs Du Laing, Karen Bradham

**Affiliations:** 1 Laboratory Microbial Ecology and Technology, Ghent University, Ghent, Belgium; 2 Microbiological and Chemical Exposure Assessment Research Division, National Exposure Research Laboratory, Office of Research and Development, U.S. Environmental Protection Agency, Cincinnati, Ohio, USA; 3 School of Environment and Natural Resources, The Ohio State University, Columbus, Ohio, USA; 4 Laboratory Analytical and Applied Ecochemistry, Ghent University, Ghent, Belgium; 5 National Exposure Research Laboratory, Office of Research and Development, U.S. Environmental Protection Agency, Research Triangle Park, North Carolina, USA

**Keywords:** arsenic species, bacteria, colon, gastrointestinal, metalloid, microflora, presystemic metabolism, Simulator of the Human Intestinal Microbial Ecosystem, speciation

## Abstract

**Background:**

Speciation analysis is essential when evaluating risks from arsenic (As) exposure. In an oral exposure scenario, the importance of presystemic metabolism by gut microorganisms has been evidenced with *in vivo* animal models and *in vitro* experiments with animal microbiota. However, it is unclear whether human microbiota display similar As metabolism, especially when present in a contaminated matrix.

**Objectives:**

We evaluated the metabolic potency of *in vitro* cultured human colon microbiota toward inorganic As (iAs) and As-contaminated soils.

**Methods:**

A colon microbial community was cultured in a dynamic model of the human gut. These colon microbiota were incubated with iAs and with As-contaminated urban soils. We determined As speciation analysis using high-performance liquid chromatography coupled with inductively coupled plasma mass spectrometry.

**Results:**

We found a high degree of methylation for colon digests both of iAs (10 μg methylarsenical/g biomass/hr) and of As-contaminated soils (up to 28 μg/g biomass/hr). Besides the formation of monomethylarsonic acid (MMA^V^), we detected the highly toxic monomethylarsonous acid (MMA^III^). Moreover, this is the first description of microbial thiolation leading to monomethylmonothioarsonic acid (MMMTA^V^). MMMTA^V^, the toxicokinetic properties of which are not well known, was in many cases a major metabolite.

**Conclusions:**

Presystemic As metabolism is a significant process in the human body. Toxicokinetic studies aiming to completely elucidate the As metabolic pathway would therefore benefit from incorporating the metabolic potency of human gut microbiota. This will result in more accurate risk characterization associated with As exposures.

Arsenic (As), a ubiquitous environmental contaminant, presents significant human health risks: Chronic exposure is associated with the development of cancer in the bladder, liver, kidney, and lungs (Chen et al. 1992). Regions with a high geogenic As background show an increased risk for elevated exposure by consumption of drinking water and diet. An additional exposure scenario in urban areas near smelting and mining activities is the ingestion of contaminated soil and dust by children, who display typical hand-to-mouth behavior. Although inorganic As (iAs) may be the predominant form in contaminated soils, As speciation changes during gastrointestinal transit are not well characterized. The gut represents a highly reducing environment and harbors a complex microbial community, which may contribute to the presystemic biotransformation of ingested As (systemic metabolism being defined as all metabolic reactions carried out by human cells). Presystemic As speciation analysis must therefore be considered an essential part of the risk evaluation process, especially with respect to toxicity, which is speciation dependent. In short, methylated trivalent species—monomethylarsonous acid (MMA^III^), dimethylarsinous acid (DMA^III^), and arsenous acid (iAs^III^)—are two orders of magnitude more cytotoxic than is As acid (iAs^V^) (Naranmandura et al. 2007a). The methylated pentavalent species—monomethylarsonic acid (MMA^V^) and dimethylarsinic acid (DMA^V^)—present a 10-fold lower toxicity than iAs^V^, whereas trimethylarsine oxide (TMAO) is essentially nontoxic (Hirano et al. 2004).

In the human body, iAs is sequentially methylated and predominately excreted as DMA^V^ in urine. This methylation process was originally considered a detoxification process, but the formation of reactive intermediates (MMA^III^ and DMA^III^) has forced researchers to reconsider methylation as an activation process (Styblo et al. 2002). In addition, a recent study on human urine analysis after iAs exposure revealed new sulfur-containing methylated As metabolites, monomethylmonothioarsonic acid (MMMTA^V^) and dimethylmonothioarsinic acid (DMMTA^V^) (Naranmandura et al. 2007b; Raml et al. 2007), for which the mechanism of formation and toxicological profile are not yet fully characterized. Given the toxicological importance of As speciation changes, it is clear that a complete risk characterization after As exposure must include the possibility of presystemic metabolism by the microbe-rich environment of the gastrointestinal tract.

The colon harbors a vast (10^14^ bacterial cells) and incredibly diverse (> 1,000 species) microbial community, which has the ability to metabolize xenobiotics far more extensively than any other part of the body (Sousa et al. 2008). Thus far, the presystemic biotransformation of As was primarily studied with gut microbiota from animal models. Rowland and Davies (1981) reported the reduction of iAs^V^ to iAs^III^ by rat cecal bacteria as well as limited formation of MMA and DMA. In another study with rats orally exposed to DMA^V^ Chen et al. (1996) detected demethylated (iAs^V^, MMA^V^) and methylated (TMAO) urinary metabolites. Finally, the thiolation of methylated As oxides (DMA^V^, TMAO) in the cecal contents of a mouse (Kubachka et al. 2009a, 2009b) and the observed thiolation through *in vivo* experiments (Kuroda et al. 2004; Naranmandura et al. 2007b) have been reported.

Presystemic As metabolism in the human body has been less investigated (Hirner et al. 2004). Nevertheless, Michalke et al. (2008) reported that human gut microbes actively volatilize bismuth and other metalloids, including As, through methylation and hydrogenation. Moreover, Meyer et al. (2008) postulated that gut methanogens play a crucial role in metalloid volatilization, thereby exerting toxic effects to the human body—not only by direct interaction with the host but also by disturbing the endogenous gut microbiota composition and metabolism. Finally, a thorough *in vitro* exploration with the Simulator of the Human Intestinal Microbial Ecosystem (SHIME), a dynamic human gastrointestinal simulator, revealed a high microbial metabolic potency toward metal(loid)s (Diaz-Bone and Van de Wiele 2009). This was demonstrated by the finding of significant volatilization of As, selenium, bismuth, tellurium (Te), and antimony; the formation of highly toxic AsH_3_ (arsine) and (CH_3_)_2_Te (dimethyl telluride); and the discovery of two new As–sulfur metabolites.

These data indicate the need for more studies with human gut microorganisms, which can confirm the presystemic metabolism as observed with animal gut microbiota. Therefore, in the present study we investigated the metabolic potency of human gut microorganisms toward iAs and As from contaminated urban soils, assessing the importance of presystemic As biotransformation upon an oral exposure scenario and the actual speciation of As that enters the bloodstream upon gastrointestinal digestion.

## Materials and Methods

### Chemicals and media

We used degassed, ultrapure 18 mΩ water (DDI; Millipore, Bedford, MA, USA) to prepare the chromatographic mobile phase and the standard stock solutions. American Chemical Society–grade ammonium nitrate and ammonium dihydrogen phosphate (Fisher Scientific, Pittsburgh, PA, USA) and technical-grade EDTA, tetrasodium salt dehydrate (Fisher Scientific, Fair Lawn, NJ, USA) were used in the chromatographic mobile phase. We obtained stock solutions of iAs (As^III^ and As^V^) from Spex Industries (Metuchen, NJ, USA) and certified stock solutions of MMA^V^ and DMA^V^ from Chem Service (West Chester, PA). W.R. Cullen (Department of Chemistry, University of British Columbia, Vancouver, BC, Canada) provided tetramethyl-cyclo-tetraarsaoxane [cyclo-(CH_3_AsO)_4_] crystals that were synthesized and characterized as described elsewhere (Cullen et al. 1989); these crystals were stored at −21°C and were hydrolyzed by degassed, deionized water at the time of analysis to obtain a stock solution of a MMA^III^ and MMA^V^ mixture (Cullen et al. 1989). We purchased sodium arsenate (Na_2_HAsO_4_·7H_2_O), methionine, methylcobalamine, and glutathione from Sigma-Aldrich (St. Louis, MO, USA). Arsenate stock solutions were prepared in deionized water at 4,500 mg As/L and 45 mg As/L.

### Soils

The U.S. Environmental Protection Agency kindly provided four As-contaminated soils that originated from urban areas around former smelting sites. We sieved all soils at 250 μm before *in vitro* gastrointestinal incubation; this sieving reflects the size of particles that most likely sticks to the hands of exposed humans (Kelly et al. 2002). Soil specifications are reported in Table 1.

### Production and characterization of colon microbiota for SHIME

The *in vitro* colon microbial community used in this study was cultured and maintained in a modified SHIME, which consisted of four compartments simulating the stomach, small intestine, and both proximal and distal colon. A detailed description of the SHIME, the carbohydrate-based medium, and the *in vitro* colon microbiota has been described previously (Van de Wiele et al. 2004). Briefly, fecal microbiota previously obtained from a 29-year-old male volunteer (who had no history of antibiotic treatment in the 6 months before the study) were inoculated in the different colon compartments. The SHIME reactor was fed carbohydrate-based medium three times per day to provide digested nutrition for the colon microbiota. After 3 weeks of adaptation, a stable microbial community was obtained in the respective colon compartments. We found microbial fermentation activity of the distal colon (short-chain fatty acid production and ammonium production) and community composition to be consistent with that of previous SHIME runs and the *in vivo* situation (Molly et al. 1994; Van de Wiele et al. 2004) [see Supplemental Material, Table 1 (doi:10.1289/ehp.0901794)].

### Noncontinuous incubation studies

#### Metabolic potency of fecal microbial inoculum

The first experiment constituted a screening phase to test whether the fecal microbial community from human origin actively metabolized As. The microbial community was isolated from a fecal sample as previously described by Molly et al. (1994). Thirty milliliters of microbial fecal suspension was sampled, placed in 60-mL serum bottles, and incubated with NaH_2_AsO_4_·7H_2_O (iAs^V^; 90 mg iAs^V^/L), similar to the method of Herbel et al. (2002). Serum bottles were capped with butyl rubber stoppers that are impervious to O_2_ and subsequently made anaerobic by flushing with N_2_ gas for 30 min. Samples were then incubated at 37°C on a rotary shaker (150 rpm) for 48 hr. We compared the effect of specific methyl group donors toward microbial As methylation by comparing methionine-amended (5 mmol/L) and methylcobalamin-amended (5 mmol/L) samples with control samples (incubation of the sample in the presence of heat-sterilized fecal microbiota). The effect of glutathione as a reducing agent was evaluated by comparing glutathione-amended samples (10 mmol/L) with control samples. Duplicate incubations were performed on two different days to evaluate the reproducibility. A scheme of the experimental setup is presented in Supplemental Material, Figure 1 (doi:10.1289/ehp.0901794).

#### Metabolic potency of colon microbiota toward As from contaminated soils

The objective of the second experiment was to screen for microbial speciation changes of iAs^V^ at more relevant concentrations (i.e., 50–500 μg/L) by mimicking conditions of oral exposure to environmental samples. In addition, four As-contaminated soil samples (one slag soil and three from urban sites) were subjected to a gastrointestinal digestion procedure. To better mimic *in vivo* conditions, all gastrointestinal stages—gastric, small intestine, and colon—were simulated. We combined the *in vitro* gastrointestinal method (IVG) from Ohio State University with the SHIME to subsequently simulate stomach and small intestine (IVG) and colon (SHIME) conditions, respectively. The IVG method was previously validated against *in vivo* data for As bioaccessibility (Rodriguez and Basta 1999), whereas the SHIME has been validated against *in vivo* data for microbial community composition and metabolic activity toward drugs and phytoestrogens (Molly et al. 1994; Possemiers et al. 2006).

Soils were incubated in the gastric and intestine solution (30 mL) of the IVG protocol at a liquid-to-soil (L/S) ratio of 150 (Rodriguez and Basta 1999). These intestinal digests from the IVG protocol were subjected to colon conditions by adding 30 mL of the colon suspension sampled from the distal colon compartment of the SHIME reactor, resulting in an L/S ratio of 300 for the soil digests. The vessels containing the colon digests were capped with butyl rubber stoppers and subsequently flushed with N_2_ for 30 min to obtain anaerobic conditions and incubated on a shaker at 150 rpm at 37°C for 18 hr. See Supplemental Material, Figure 1 (doi:10.1289/ehp.0901794) for a schematic of the experimental setup.

### Sample treatment

To preserve the speciation of As in the colon digests, all samples were flash frozen with liquid nitrogen upon incubation and subsequently stored at −80°C. Before analysis with high-performance liquid chromatography (HPLC) coupled with inductively coupled plasma (ICP) mass spectrometry (MS), samples were thawed and diluted appropriately with 20 mmol/L (NH_4_)_2_CO_3_ at pH 9.0 to minimize sulfur–oxygen exchange while awaiting analysis (Conklin et al. 2008). Upon complete thawing, the sample was vortexed and centrifuged for 10 min at 10,400 relative centrifugal force with an Eppendorf 5810R centrifuge (Brinkman Instruments, Westburg, NY, USA) to separate soluble As species from soil-bound As. The supernatant was filtered through a Millex-LCR 0.45 μm filter (Millipore) with a Luer-Lok 10-mL syringe (BD, Franklin Lakes, NJ, USA). Finally, filtrates were diluted with the mobile phase and injected into the HPLC. The sum of the As species in the filtrate observed chromatographically was considered the bioaccessible fraction. We measured total As concentration in the digest filtrates using ICP optical emission spectroscopy (ICP-OES). This allowed us to calculate chromatographic recovery, which quantifies the extent to which the sum of the chromatographic As species comprises the total amount of As in the digest filtrates.

### As speciation analysis by HPLC/ICP-MS

Sample supernatants were analyzed with HPLC (Agilent 1100) and ICP-MS (Agilent 7500ce; Agilent, Palo Alto, CA, USA) for As specific detection at *m*/*z* 75. Separation of As oxides was performed on a PRP-X100 HPLC column (250 mm × 4.1 mm, 5 μm). The mobile phase was a solution of NH_4_NO_3_ (10 mmol/L), NH_4_H_2_PO_4_ (10 mmol/L), and EDTA (500 mg/L) at pH 4.57 in distilled water (separation 1). The flow rate was 1.0 mL/min, and the sample injection volume was 100 μL. The retention times of the separated compounds were 3.6 min for As^III^, 4.2 min for DMA^V^, 5.5 min for MMA^III^, 7.1 min for MMA^V^, and 8.9 min for As^V^, similar to those previously reported by Yathavakilla et al. (2008). We used this separation for quantification of the As species of interest. Arsenic sulfides were identified by retention-time matching between samples and fortified samples. Using chromatographic separation 1, monothioarsonic acid eluted at 15 min, whereas MMMTA^V^ eluted at 18.6 min. See the Supplemental Material (doi:10.1289/ehp.0901794) for details on synthesis, chromatographic confirmation of these As sulfides, and sample analysis.

A second chromatography [separation 2; see Supplemental Material (doi:10.1289/ehp.0901794)] was used for ICP-MS and electrospray ionization (ESI)-MS detection, because the mobile phase of separation 1 was not compatible with ESI-MS detection.

## Results

In the first experiment, we assessed the metabolic potency of the human fecal microbial inoculum toward high levels of iAs^V^ (90 mg/L). iAs^V^ was efficiently (> 94%) reduced to iAs^III^ after the 48-hr incubation with both active and sterilized fecal microbiota (Table 2), probably because of the highly reducing conditions (redox potential was −180 mV). Incubation with sterilized fecal microbiota did not lead to thiolated or methylated arsenicals. In contrast, incubation of iAs^V^ with active fecal microbiota resulted in the production of monothioarsonic acid (mTA; mean ± SD) in nonamended (2.2 ± 3.1 mg/L) and methionine-amended (0.8 ± 1.1 mg/L) samples. Interestingly, we observed methylation only in the presence of methylcobalamin. Addition of the methylcobalamin displayed a significant methylation of iAs (18%), with MMA^V^ (13.0 ± 1.4 mg/L) being more dominant than MMA^III^ (2.6 ± 1.4 mg/L). The addition of both methylcobalamin and glutathione as a reducing agent increased the methylation to 28%, with MMA^III^ (10.5 ± 5.4 mg/L) becoming equally as important as MMA^V^ (11.3 ± 5.6 mg/L).

These preliminary data convinced us that the selected microbial community had the potency to actively metabolize iAs^V^. We therefore inoculated the SHIME reactor with this fecal microbial inoculum; after 3 weeks of adaptation, a stable microbial community was obtained in the proximal and distal colon compartments. We regularly sampled the distal colon compartment to perform colon incubations on iAs^V^ and As-contaminated soil samples that had already gone through a gastric and intestinal digestion. Characterization of the colon digests consisted of determining As bioaccessibility and As speciation. The bioaccessibility determination was based on the sum of all chromatographically detected (HPLC/ICP-MS) As species in the filtrates (0.45 μm) of the colon digests; therefore, the chromatographic recovery was calculated first. The sum of the concentrations of chromatographically detected As species in the colon filtrates was divided by the total As concentration in the colon filtrates, as measured by ICP-OES. The chromatographic recoveries for all colon digests, except for that of soil 4, were satisfactorily high: 93 ± 7% (mean ± SD) on average [the recovery of the soil 4 digest excluded; see Supplemental Material, Table 2 (doi:10.1289/ehp.0901794)]. Hence, most As species present in these digest supernatants could be detected with the HPLC/ICP-MS protocol. Bioaccessibility calculations for these digests displayed the highest value (75.5%) for the iAs^V^-incubated colon digest, whereas colon incubation of the contaminated soils resulted in As bioaccessibility values of 24% (soil 1), 44% (soil 2), and 36% (soil 3) (Table 3). In sharp contrast, As bioaccessibility in the colon digest of soil 4 was only 0.3%. Even when taking into account the low chromatographic recovery of 15%, we obtained a low As bioaccessibility of 2.4%, which is still an order of magnitude lower than the bioaccessibility values for the other soil digests. Overall, colon bioaccessibility values (Table 3) for the four soils were consistently lower than the corresponding intestinal bioaccessibility values (Table 1) obtained with the IVG method.

**Figure 1:**
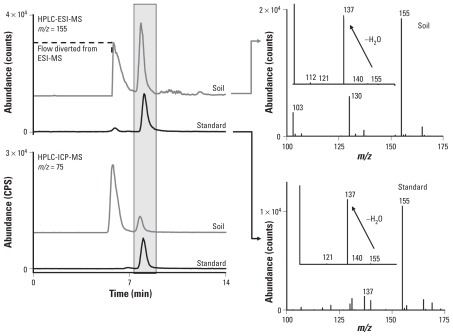
Identification of MMMTA^V^ by HPLC/ESI-MS/MS and HPLC/ICP-MS (left) [using separation 2; see Supplemental Material (doi:10.1289/ehp.0901794)]. The gray and black traces in chromatograms represent analysis of a soil extract and MMMTA^V^ standard, respectively. Right: MS/MS spectra of *m*/*z* 155 within each MS spectra.

The most important part of this study consisted of the As speciation analysis of the colon digests after the gastrointestinal incubation of iAs^V^ and the four contaminated soils. The original analytical protocol was optimized to detect the presence of iAs^III^, iAs^V^, MMA^III^, and MMA^V^. We detected an additional As species, MMMTA^V^, in many of the colon digests. We initially identified MMMTA^V^ using a combination of retention-time matching and by fortifying the sample with the suspected standard using separation 1 with ICP-MS detection, but we used a second chromatography (separation 2) for ICP-MS and ESI-MS detection. Figure 1 shows HPLC/ICP-MS mass chromatograms of *m*/*z* 75 (^75^As) and HPLC/ESI-MS mass chromatograms of *m*/*z* 155 ([M-H]^−^) for an MMMTA^V^ standard and a SHIME extract using separation 2. The retention times of the MMMTA^V^ in the standard and MMMTA^V^ in the sample were slightly offset because the matrix of the soil extract caused the decreased retention of MMMTA^V^ on the C_18_ column. Tandem MS (MS/MS) of *m*/*z* 155 yielded a product ion of *m*/*z* 137 (loss of H_2_O) and, to a lesser extent, a product ion of *m*/*z* 121 (due to CH_2_AsO_2_^−^) and *m*/*z* 140 (loss of CH_3_). The molecular mass of 155 and corresponding fragments were consistent with other reports for MMMTA^V^ (Yathavakilla et al. 2008).

**Figure 2:**
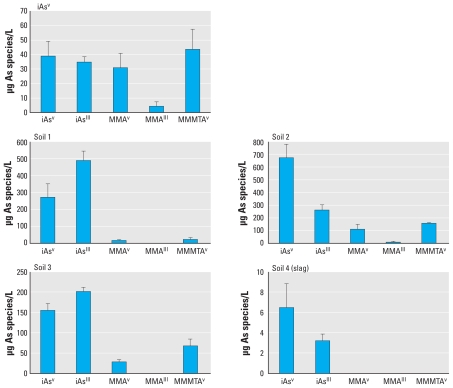
Concentration (mean ± SD) of chromatographically detected As species in colon digests of iAs^V^ (225 μg/L) and of As-contaminated soils 1–4 (*n* = 3). Note the different scales for the As concentrations (*y*-axis) for iAs^V^ and contaminated soils.

We detected significant As methylation upon colon incubation of 225 μg iAs^V^/L (Figure 2). The sum of the concentrations of MMA^V^ (31.0 μg/L), MMA^III^ (4.5 μg/L), and MMMTA^V^ (43.7 μg/L) exceeded that of iAs^V^ (39.0 μg/L) and iAs^III^ (34.8 μg/L). In contrast, iAs species were predominantly present in colon digests of soils 1, 2, and 3, whereas they were the only As species in the colon digest of slag soil 4 (Figure 2). The colon digest of soil 1 displayed a methylation percentage of 4.7% with MMA^V^ (17 μg/L) and MMMTA^V^ (23 μg/L) as detected methylarsenicals. The methylation percentage for colon digests of soil 2 (22.8%) and soil 3 (21.2%) was higher, with soil 2 displaying MMA^V^ (111 μg/L), MMA^III^ (9 μg/L), and MMMTA^V^ (158 μg/L) and soil 3 displaying only MMA^V^ (28 μg/L) and MMMTA^V^ (68 μg/L). Finally, no methylated As species were detected in the colon digests of slag soil 4.

Summarizing the *in vitro* As speciation changes by human gut microorganisms, we calculated the specific production rates of methylated arsenicals by taking into account the initial microbial biomass and As concentrations. We obtained a methylation rate of 10 μg methylarsenicals/g biomass/hr for the colon digest of iAs^V^ (Table 3). Although no methylarsenicals were detected in colon digests of the slag soil 4, the presence of the other soil matrices did not necessarily lower the above-mentioned methylation rate. We obtained methylation rates of 4, 29, and 10 μg/g/hr for colon digests of soils 1, 2, and 3, respectively (Table 3).

## Discussion

The present study demonstrates that human colon microorganisms have the potency to actively metabolize As into methylated arsenicals and thioarsenicals, which indicates that presystemic As metabolism may not be neglected when assessing risks from oral As exposure. We observed this upon colon incubation of both iAs and As-contaminated soils. These findings parallel those from studies with animal gut microbiota (Hall et al. 1997; Rowland and Davies 1981) and suggest the existence of a presystemic As metabolism in the human body. The most important result was the detection of significant levels of MMMTA^V^ in colon digests of both iAs^V^ (25% of bioaccessible As) and of As-contaminated soils (up to 20% of bioaccessible As). To our knowledge, this is the first time that MMMTA^V^ production from iAs^V^ by human colon microbiota has been described. MMMTA^V^ production from this source resembles the methylation and thiolation of DMA^V^ into trimethylarsine sulfide by mouse cecal microbiota (Kubachka et al. 2009a) and the production of methylated thioarsenicals from DMA^V^ by rat intestinal microbiota (Kuroda et al. 2004; Yoshida et al. 2001). Yet, mammalian cells also have the ability to form methylated thioarsenicals. Kuroda et al. (2004) described rapid detection (5 min) of DMMTA^V^ and dimethyldithioarsinic acid (DMDTA^V^) after injection of of rats with DMA^III^, and Naranmandura and Suzuki (2008) reported that DMA^III^ was converted to DMDTA^V^ by human red blood cells.

The finding of presystemic MMMTA^V^ formation by human gut microorganisms raises questions about its toxicological importance. Although the absorption kinetics of MMMTA^V^ and other thiolated arsenicals across the epithelium are unknown, there is evidence that some methylated thioarsenicals elicit a higher toxicity than iAs^V^ because of their more efficient absorption by mammalian cells (Naranmandura et al. 2007a). Preliminary cytotoxicity (Naranmandura et al. 2007a; Yoshida et al. 2003) and genotoxicity (Kuroda et al. 2004) data for DMMTA^V^ show levels of toxicity similar to those of trivalent As species. Our observations in the present study emphasize the need to investigate the behavior of MMMTA^V^ in the gut lumen and the absorption rate across the intestinal epithelium. In addition, the mechanism behind the microbial production pathway needs to be elucidated. Interestingly, MMMTA^V^ levels in the colon digests correlated with those of MMA^V^ (*R*^2^ = 0.76), whereas the correlation with levels of MMA^III^ was much lower (*R*^2^ = 0.42). This seems to indicate that MMMTA^V^ in the colon digests arises from the thiolation of MMA^V^, which would correspond with earlier observations describing the interconversion between oxide and sulfide forms of MMA^V^, DMA^V^, and TMAO (Conklin et al. 2008). The sulfide source may originate from microbial sulfate reduction to hydrogen sulfide, which is a common process in the colon environment (Deplancke et al. 2000), and can trigger the formation of thioarsenosugars upon the incubation of arsenosugars with mouse cecal contents (Conklin et al. 2006). The role of sulfate-reducing microorganisms in the presystemic production pathway of MMMTA^V^ must therefore be studied further.

The significant formation of MMA^V^ and MMA^III^ after incubation of iAs^V^ with colon microorganisms was not unexpected. Arsenic methylation by rodent gut microbes (Hall et al. 1997; Rowland and Davies 1981) and human gut microbes (Diaz-Bone and Van de Wiele 2009; Meyer et al. 2008) has been described previously. Taking into account the initial biomass concentration, we observed specific methylation rates of 10 μg methylarsenicals/hr/g biomass. This roughly corresponds to 130 pmol/hr/mg biomass, which is > 16 pmol/hr/mg obtained with rat cecal microbiota (Hall et al. 1997). Interestingly, the presence of a soil matrix did not necessarily result in lower As methylation rates, yet soil-dependent parameters may have affected the methylation rate. First, comparison of the mineralogy from soils 1, 2, and 3 with that of slag (soil 4) showed an important difference in reactive iron oxide content (Table 1), which is highly efficient in sorbing As (Beak et al. 2006). The reactive iron oxide content in slag soil 4 was particularly high (18,759 μg/kg; Table 1), presumably leading to much lower As availability to colon microorganisms (0.3% bioaccessibility) and thus also limiting methylation. This observation may confirm earlier observations of slag soil mineralogy significantly decreasing As bioavailability (Davis et al. 1996). A second element in the soil-dependent As methylation may be the difference in toxic elements. Compared with the first three soils, slag (soil 4) contained high amounts of cadmium, chromium, copper, molybdenum, lead, and zinc (Table 1), which may be toxic to intestinal microorganisms. Our finding of a 70% lower fermentation activity in soil 4 colon digests versus colon digests of the other soils (data not shown) may support the assumption of slag-soil–induced toxicity. The actual relationship between gut microbial As metabolism and soil characteristics therefore needs further study.

A final aspect of our study concerns the metabolic potency of fecal microbes toward high levels of iAs^V^ (90 mg iAs^V^/L) and the influence of cofactors. Nonamended colon digests of iAs^V^ resulted in the efficient reduction to iAs^III^ and the production of mTA. Similar to the finding of MMMTA^V^, the formation of mTA may result from an oxygen-for-sulfur exchange in iAs^V^ because of the availability of sulfide, originating from the above-mentioned microbial sulfate reduction. The absence of mTA in glutathione-amended samples may be explained by the complete reduction of iAs^V^ into iAs^III^ by glutathione as reducing agent. We also evaluated the effect of methyl group donors. In contrast to methionine, methylcobalamin may be an effective methyl group donor, resulting in the efficient methylation (19%) of iAs^V^ into MMA^V^ and MMA^III^ (Table 2). The methylation efficiency increased to 25% upon cosupplementation of methylcobalamin and glutathione. We attributed this to the increased reduction of MMA^V^ into MMA^III^ by glutathione as reducing agent. In contrast to the colon digests with low levels of iAs^V^ (225 μg/L), no MMMTA^V^ was detected in the fecal digests. A probable explanation is the difference in experimental setup, the difference in microbial community composition and activity, or a difference in sulfide availability. These observations confirm a previous report that addition of cofactors may increase As methylation by enteric microorganisms, yet it is not a prerequisite for the methylation of low levels of As (micrograms per liter range) (Hall et al. 1997).

The present study provides evidence for the existence of significant presystemic As metabolism by human gut microorganisms, but the relevance for the total risk of oral As exposure is not yet clear. So far, the *in vitro* approach for assessing the risks from oral contaminant exposure mainly involved the use of models that focus on gastric and intestinal processes. Methylation of As by intestinal microorganisms was thought to contribute little to the overall methylation *in vivo* (Vahter and Gustafsson 1980) because iAs^V^ and iAs^III^ are rapidly absorbed in the small intestine (Vahter 1983), especially when As is ingested in a soluble matrix (e.g., drinking water). However, soil-bound and/or dietary-bound As may follow a different digestion scenario in the gut, and a large fraction may end up in the colon lumen, where it is subjected to the resident microbial community. The finding of MMMTA^V^ and the highly toxic MMA^III^ as metabolites from human colon microorganisms indicates that presystemic methylation will not lead to detoxification. In addition, *in vitro* studies with Caco-2 human epithelial colorectal adenocarcinoma cells suggest that the absorption of methylated arsenicals (DMA^V^, 10.0%; TMAO, 10.9%) is more efficient than that of iAs^III^ (5.8%) and iAs^V^ (1.6%) (Laparra et al. 2005, 2007). Intestinal absorption of methylated thioarsenicals should be examined in future research.

Regarding the variability between individuals regarding presystemic As metabolism, we investigated the gut microbiota from only one human. Interindividual variability in gut microbial composition is very high; thus, we expect gut microbiota from different individuals to display distinct As metabolic profiles. Such interindividual variation in metabolism by human gut microbiota was previously reported for ingested phytoestrogens (Bolca et al. 2007) and, interestingly, also for the metalloid bismuth (Michalke et al. 2008). Therefore, variability in gut microbial As metabolism should be given the same attention as the genetic variations that may govern interindividual differences in As response (Hernandez and Marcos 2008).

## Conclusion

The present study shows that presystemic metabolism of soil-derived As may be relevant in the human body when significant amounts of As become available to colon microorganisms. The absorption kinetics of methylated arsenicals and thioarsenicals across the gut epithelium and their toxicity need further elucidation. We propose that the metabolic activity of human colon microorganisms be incorporated in development of new toxicokinetic models that assess risks from oral As exposure. Mikov (1994) nicely summarized the importance of gut microbiota, stating that gut microbial metabolism must be considered an integral part of drug/xenobiotic metabolism and toxicity studies. In this context, knowledge about gut microbial metabolism must also be translated to metal(loid) biotransformation.

## Figures and Tables

**Table 1:** Characteristics and elemental composition of the four As-contaminated soils.

Characteristic	Soil 1	Soil 2	Soil 3	Soil 4 (slag)
Organic carbon (%)	5.7	4.0	3.1	2.2
pH^a^	6.1	6.3	5.0	7.2
Electric conductivity (dS/m)	0.3	0.5	0.9	0.5
Fe (mg/kg)	14,800	15,250	12,100	202,000
Mn (mg/kg)	429	525	207	1,640
Reactive Fe^b^	3,489	3,592	1,634	18,759
Percent bioaccessibility with IVG^c^	58.5	62.4	47.7	1.0
Toxic trace element content (mg/kg)				
As	990	829	379	837
Cadmium	9.7	5.5	1.5	28.9
Chromium	32.0	31.4	28.7	187
Copper	51.9	60.1	22.2	1,520
Molybdenum	2.1	2.4	1.5	73.9
Nickel	12.5	10.9	9.3	10.4
Lead	885	602	172	8,702
Selenium	330	430	127	294
Zinc	562	803	151	12,500

a Soil pH was determined in a 1:10 (wt/vol) aqueous soil slurry.

b Iron dissolved by acid ammonium oxalate extraction.

c Data from Whitacre (2009).

**Table 2:** Influence of cofactors toward microbial metabolism during *in vitro* fecal incubation of iAs^V^ at 90 mg/L.

Microbiota	As^V^	As^III^	MMA^V^	MMA^III^	mTA	Recovery (%)
Active microbiota						
No cofactor	1.7 ± 2.3	97.6 ± 2.5	ND	ND	2.2 ± 3.1	113
Me-B12	2.6	65.3	13.0 ± 1.4	2.6 ± 1.4	ND	93
Meth	1.7 ± 2.3	103.4 ± 3.1	ND	ND	0.8 ± 1.1	118
GSH	ND	93.2 ± 18.8	ND	ND	ND	104
Me-B12/Meth/GSH	2.0 ± 1.2	53.6 ± 9.7	11.3 ± 5.6	10.5 ± 5.4	ND	86
Sterilized microbiota^a^						
No cofactor	5.6	87.6	ND	ND	ND	104
Me-B12	4.6	72.7	ND	ND	ND	86
Meth	4.6	86.8	ND	ND	ND	102
GSH	ND	84.6	ND	ND	ND	94
Me-B12/Meth/GSH	3.5	83.7	ND	ND	ND	97

Abbreviations: GSH, glutathione; Me-B12, methylcobalamin; Meth, methionine; ND, not detected. Values are mean ± SD (mg/L) from duplicate incubation experiments

a Incubation tests with sterilized microbiota were performed once, so no SD is available.

**Table 3:** Percentage bioaccessibility of As and biomass-specific production rate of methylated arsenicals by colon microorganisms after *in vitro* colon digestion of iAs^V^ (225 μg/L) and As-contaminated soils.

Sample	Percent bioaccessibility^a^	Methylation rate (μg/hr/g biomass)^b^
Na_2_HAsO_4_·7H_2_O	76 ± 14.3	10.0 ± 4.0
Soil 1	24 ± 4.3	10.0 ± 1.8
Soil 2	44 ± 3.9	4.2 ± 1.6
Soil 3	36 ± 1.5	28.9 ± 3.4
Soil 4	0.3 ± 0.1	ND

ND, not detected. Values are mean ± SD of three experiments.

a Calculated by dividing the sum of detected As species (iAs^V^, iAs^III^, mTA, MMA^V^, MMA^III^, and MMMTA^V^) in the colon digest filtrate by the total amount of As that was incubated under colon conditions.

b Calculated by dividing the sum of methylated arsenicals (MMA^V^, MMA^III^, and MMMTA^V^) in the colon digest filtrate by the initial biomass concentration and the incubation time.
